# Residual Sleep-Related Respiratory Events During Early Versus Long-Term NIV Follow-Up: Burden and Relationship with Gas Exchange

**DOI:** 10.3390/jcm15197695

**Published:** 2026-10-04

**Authors:** Pierre Tankéré, Marjolaine Georges, Ameline Vagner, Jade Chorvoz, Geoffroy Mery, Cedric Bongard, Philippe Bonniaud, Claudio Rabec

**Affiliations:** 1 Department of Pulmonology, Croix-Rousse Hospital, Hospices Civils de Lyon, 69004 Lyon, France; 2 Research on Healthcare Performance (RESHAPE), INSERM U1290, University Claude Bernard Lyon 1, 69373 Lyon, France; 3 Hypoxie Physiopathologie (HP2), INSERM U1300, University Grenoble Alpes, 38058 Grenoble, France; 4 Institut Universitaire du Poumon, Centre de Référence Constitutif des Maladies Pulmonaires Rares de l’Adulte, Centre Hospitalo-Universitaire Dijon-Bourgogne, 21000 Dijon, France; 5 Faculty of Medicine and Pharmacy, University Bourgogne Europe, 21000 Dijon, France; 6 Centre des Sciences du Goût et de l’Alimentation, INRAE, UMR 6265 CNRS 1234, University Bourgogne Europe, 21000 Dijon, France; 7 Division of Pulmonary Medicine, Lausanne University Hospital (CHUV), University of Lausanne, CH-1011 Lausanne, Switzerland; 8 Center for Translational and Molecular Medicine (CTM), INSERM U1231, University Bourgogne Europe, 21000 Dijon, France

**Keywords:** NIV, non-invasive ventilation, chronic respiratory failure, patient–ventilator asynchrony

## Abstract

**Background:** A recent study published by our group demonstrated that patient–ventilator asynchrony (PVA) is independently associated with impaired gas exchange during long-term non-invasive ventilation (NIV). We aimed to determine whether the burden and phenotype of residual PVA vary according to disease category, NIV initiation context and follow-up timing, and whether the relationship between PVA and gas exchange differs according to follow-up timing. **Methods:** We performed a secondary analysis of 123 NIV-treated patients included in the primary study. PVA burden, upper-airway obstructive events and leaks were compared across disease categories and follow-up settings. Multivariable models for gas exchange outcomes were extended by introducing interaction terms between PVA burden and follow-up category. Patients underwent either long-term follow-up performed >3 months after NIV initiation (*n* = 74), early acute follow-up, i.e., after stabilization following NIV initiation in acute respiratory failure (*n* = 30), or early planned follow-up, i.e., within 3 months after NIV initiation in stable chronic respiratory failure (CRF) (*n* = 19). **Results:** Overall, leaks, upper airway obstructions (UAOs) and PVA burden did not significantly differ across disease categories, and neither leaks nor UAO differed across follow-up categories. In contrast, patients undergoing early follow-up after acute NIV initiation exhibited a significantly greater burden of residual PVA than those initiated in stable CRF, predominantly because of ineffective efforts. Within the subgroup of patients undergoing early follow-up, total PVA burden was more than doubled after acute NIV initiation (4.5% versus 1.5% of recording time; 6.2 versus 2.5 events·h^−1^). The association between PVA burden and post-NIV PaCO_2_ did not differ according to follow-up timing. Conversely, in an exploratory analysis limited to 40 patients, the relationship between PVA burden and nocturnal TcPCO_2_ was significantly attenuated during early compared with long-term follow-up (interaction β = −1.04, 95% CI −1.68 to −0.40; *p* = 0.003). However, this interaction was attenuated after adjustment for daytime PaCO_2_ (β = −0.55, 95% CI −1.12 to 0.03; *p* = 0.076). Stratified analyses demonstrated significant associations between PVA burden and both post-NIV PaCO_2_ (β = 0.38, *p* = 0.034) and nocturnal TcPCO_2_ (β = 0.80, *p* < 0.001) during routine long-term follow-up. **Conclusions:** Patients evaluated after acute NIV initiation exhibited the highest burden of residual PVA, predominantly ineffective efforts. However, PVA was independently associated with nocturnal hypercapnia primarily during established long-term NIV. These findings should be considered hypothesis-generating and require confirmation in larger prospectively monitored cohorts.

## 1. Background

Patient–ventilator asynchrony (PVA) is increasingly recognized as a major determinant of non-invasive ventilation (NIV) effectiveness. During long-term NIV, residual respiratory events include unintentional leaks, upper-airway obstructive events and patient–ventilator asynchronies (PVAs), including rate asynchronies, such as ineffective efforts, double triggering and auto triggering. To facilitate standardized assessment of these events, Gonzalez-Bermejo et al. proposed a dedicated framework for the classification of PVA during long-term NIV [1]. More recently, Longhini et al. reviewed the challenges associated with detecting and monitoring patient–ventilator asynchrony during both acute and stable CRF NIV, highlighting the continuing need for standardized assessment and clinically meaningful outcome measures [2].

Several studies have shown that residual respiratory events remain common despite apparently adequate ventilator settings. In patients receiving NIV for acute respiratory failure, asynchronies are frequent and are strongly influenced by leaks and ventilator settings [3,4]. In contrast, studies performed after clinical stabilization during long-term NIV have reported a predominance of ineffective efforts during sleep, particularly in patients with COPD and neuromuscular disorders [5,6,7,8]. Most available studies have evaluated PVA either during acute NIV or during established long-term NIV, making it difficult to determine whether the burden and phenotype of residual asynchronies evolve after NIV initiation.

However, the clinical significance of these residual events remains uncertain. Although previous work suggested an association between patient–ventilator asynchrony and impaired nocturnal ventilation during NIV titration [9], Aarrestad et al. failed to demonstrate a clear relationship between respiratory events and gas exchange during long-term NIV in stable patients [10]. Furthermore, little is known about whether the physiological consequences of residual asynchronies differ according to the context of NIV follow-up.

A similar knowledge gap exists in pediatric NIV. Polygraphic studies have demonstrated that residual respiratory events and patient–ventilator asynchronies remain common during long-term NIV in children and may affect sleep quality and ventilatory efficacy [11]. More recently, Collignon et al. showed that asynchronies may significantly increase the work of breathing in ventilated children, highlighting the potential physiological consequences of persistent patient–ventilator asynchronies [12].

In a recent study, we demonstrated that patient–ventilator asynchrony is independently associated with impaired gas exchange during long-term NIV [13]. The present analysis was designed to determine whether this relationship varies according to disease category and more importantly, according to the timing and the clinical context of NIV follow-up.

## 2. Methods

This study was conducted as a secondary analysis of the cohort previously described in our investigation of sleep-related respiratory events during long-term non-invasive ventilation (NIV) [13]. All patients underwent overnight polygraphy with manual scoring of patient–ventilator asynchronies (PVAs), upper-airway obstructive events and unintentional leaks according to the methodology reported in the primary study.

The objectives of the present analysis were twofold. First, we sought to determine whether the burden and phenotype of PVA differed according to the underlying cause of CRF and according to the clinical context in which NIV was evaluated. Second, we investigated whether the physiological impact of PVA on gas exchange differed according to the timing of follow-up.

Patients were classified according to their underlying disease as chronic obstructive pulmonary disease (COPD), neuromuscular disease (NMD), obesity hypoventilation syndrome (OHS) or restrictive thoracic disorders (RTD). In addition, patients were classified into three follow-up categories according to the timing and context of NIV initiation: (1) early follow-up after acute NIV initiation (hereafter referred to as the “early acute follow-up” group), (2) early follow-up after planned NIV initiation (“early planned follow-up” group), and (3) follow-up performed more than 3 months after NIV initiation during established long-term NIV treatment (“long-term follow-up” group).

Early follow-up assessments were performed after clinical stabilization, on the night before hospital discharge and carried out in the dedicated ventilation unit. Accordingly, none of the evaluations were conducted during acute respiratory failure. The timing of those assessments reflected routine clinical practice and was determined by the treating physician according to the patient’s clinical course following NIV initiation.

When prescribed, long-term oxygen therapy was used in addition to NIV for an independent indication of chronic hypoxemia or for hypoxemia persisting despite optimization of ventilatory support, according to French eligibility criteria. Analyses of mean nocturnal SpO_2_ were restricted to patients not receiving supplemental oxygen.

Respiratory events were quantified both as event indices (events·h^−1^) and as the percentage of recording time spent in each event category. Total PVA burden, ineffective efforts, double triggering, autotriggering, upper-airway obstructive events with and without inspiratory effort and unintentional leaks were compared across disease categories and follow-up settings. Baseline demographic, clinical and ventilatory characteristics were compared across follow-up categories to identify potential differences in patient profiles and NIV management.

To place these observations in the context of our previous findings, we subsequently extended the multivariable models developed in the primary study [13]. Interaction terms between PVA burden and follow-up category were introduced to evaluate whether the association between PVA and gas exchange outcomes differed according to follow-up category. Thus, each model included the main effects of PVA burden and follow-up category, together with their interaction term (PVA burden × follow-up category). A significant interaction was interpreted as suggesting that the association between PVA burden and the outcome varied according to follow-up category. Outcomes included post-NIV arterial carbon dioxide tension (PaCO_2_), mean nocturnal transcutaneous carbon dioxide tension (TcPCO_2_) and mean nocturnal oxygen saturation (SpO_2_). A sensitivity analysis additionally adjusted the TcPCO_2_ interaction model for PaCO_2_. A separate exploratory analysis restricted to early follow-up compared acute and planned NIV initiation using two-sided *t*-tests. Models were adjusted for disease category, upper-airway obstructive events and unintentional leaks. Results are reported as regression coefficients (β), 95% confidence intervals (95% CIs) and two-sided *p*-values. During the preparation of this manuscript, the authors used artificial intelligence exclusively for English language editing, grammar, and readability (ChatGPT-5.6, OpenAI, San Francisco, CA, USA).

## 3. Results

### 3.1. Patient Characteristics According to Follow-Up Category

Baseline characteristics according to follow-up category are presented in Table 1. Time since NIV initiation averaged 38.2 ± 52.1 months in the long-term follow-up group. Patients evaluated shortly after acute NIV initiation tended to be older than those undergoing long-term follow-up (71.1 ± 14.5 versus 63.2 ± 17.7 years, *p* = 0.054) and were more frequently female (73.3% versus 45.9%, *p* = 0.040). Disease distribution, body mass index, pulmonary function and the prevalence of obstructive sleep apnea were otherwise broadly comparable across follow-up categories.

Patients undergoing early follow-up after acute NIV initiation were more likely to receive long-term oxygen therapy than those evaluated after early planned NIV initiation (60.0% versus 26.3%, *p* = 0.047). Ventilator settings were generally similar across groups, although inspiratory pressure support was lower in the early planned follow-up group (9.9 ± 2.6 cmH_2_O versus 11.7 ± 2.5 and 12.0 ± 3.4 cmH_2_O, *p* = 0.037). Daytime PaCO_2_ differed significantly according to follow-up category, averaging 54.8 ± 8.7 mmHg after acute NIV initiation, 52.8 ± 8.1 mmHg after planned NIV initiation and 47.2 ± 8.6 mmHg during long-term follow-up (*p* < 0.001).

### 3.2. Residual Sleep-Related Respiratory Events According to Disease Category

A total of 123 patients were included. The overall burden of sleep-related respiratory events remained relatively similar across the major causes of CRF. Total patient–ventilator asynchrony (PVA), upper-airway obstructive events and unintentional leaks did not significantly differ between patients with chronic obstructive pulmonary disease (COPD), neuromuscular disease (NMD), obesity hypoventilation syndrome (OHS) and restrictive thoracic disorders (RTD), whether expressed as percentage of recording time or as event indices (Table 2).

Analysis of specific PVA subtypes showed some heterogeneity in event composition. Restrictive thoracic disorders tended to exhibit the highest burden of ineffective efforts, whereas obesity hypoventilation syndrome showed higher levels of double triggering. However, none of these differences reached statistical significance, and no statistically significant differences were observed when these events were expressed as event indices. Overall, no statistically significant differences in residual respiratory events were detected across disease categories, although limited subgroup sizes preclude firm conclusions of equivalence or independence from underlying disease (Table 2).

### 3.3. Residual Sleep-Related Respiratory Events According to Follow-Up Context

Among the 123 included patients, 74 underwent routine long-term follow-up during established NIV treatment (“long-term follow-up” group), 19 underwent early follow-up after NIV initiation in stable CRF (“early planned follow-up” group), and 30 underwent early follow-up after NIV initiation for acute respiratory failure (“early acute follow-up” group).

Patient–ventilator asynchronies differed significantly according to follow-up context, whereas leaks and upper-airway obstructive events remained comparable between groups (Table 3). Patients evaluated after acute NIV initiation exhibited the highest burden of residual PVA. Total PVA burden reached 5.7% of recording time and 8.9 events·h^−1^ in the early acute follow-up group, compared with 3.4% and 3.9 events·h^−1^ in the early planned follow-up group and 3.7% and 4.0 events·h^−1^ during long-term follow-up. Differences across follow-up categories were significant whether PVA burden was expressed as a percentage of recording time (*p* = 0.006) or as an event index (*p* = 0.007).

This difference was mainly accounted for by ineffective efforts, which reached 4.9% of recording time and 7.2 events·h^−1^ in the acute follow-up group, compared with 1.4% and 1.8 events·h^−1^ in the early planned follow-up group and 3.0% and 3.1 events·h^−1^ during routine long-term follow-up. Differences across follow-up categories were significant both when expressed as a percentage of recording time (*p* = 0.006) and as an event index (*p* = 0.011).

By contrast, upper-airway obstructive events, leaks and double triggering did not significantly differ between follow-up groups. Autotriggering also differed significantly across follow-up groups, although it represented only a minor component of the overall residual respiratory-event burden (<1.5 events·h^−1^ in all groups) (Table 3).

In a separate exploratory analysis restricted to the 49 patients undergoing early follow-up, median total PVA burden was higher after acute than planned NIV initiation: 6.15 (0.30–16.00) versus 2.50 (0.65–4.40) events·h^−1^ (*p* = 0.02). Ineffective efforts were also significantly more frequent after acute initiation when expressed as a percentage of recording time. No statistically significant differences were detected for double triggering, autotriggering, leaks, or upper-airway obstructive events. *p*-values were obtained using two-sided *t*-tests comparing group means. These findings indicate that PVA, rather than leaks or upper-airway obstructive events, was the only residual respiratory event varying according to follow-up timing (Table 3). We therefore focused subsequent analyses on the relationship between PVA burden and gas exchange outcomes according to follow-up timing.

### 3.4. Relationship Between Patient–Ventilator Asynchronies and Gas Exchange

Post-NIV PaCO_2_ was available in 122/123 patients (99.2%), mean nocturnal SpO_2_ in all patients (123/123), whereas nocturnal TcPCO_2_ was available in 40/123 patients (32.5%), including 7/30 (23.3%) in the early acute group, 7/19 (36.8%) in the early planned group and 26/74 (35.1%) in the long-term follow-up group. Compared with the 83 patients without TcPCO_2_ data, no statistically significant differences were observed in follow-up category, demographic characteristics, underlying disease, gas exchange, ventilator settings, or residual respiratory-event burden (Supplementary Table S1).

Despite the higher burden of residual asynchrony observed after acute NIV initiation, an exploratory interaction was observed between follow-up timing and the association between PVA burden and nocturnal hypercapnia.

**Figure 1:**
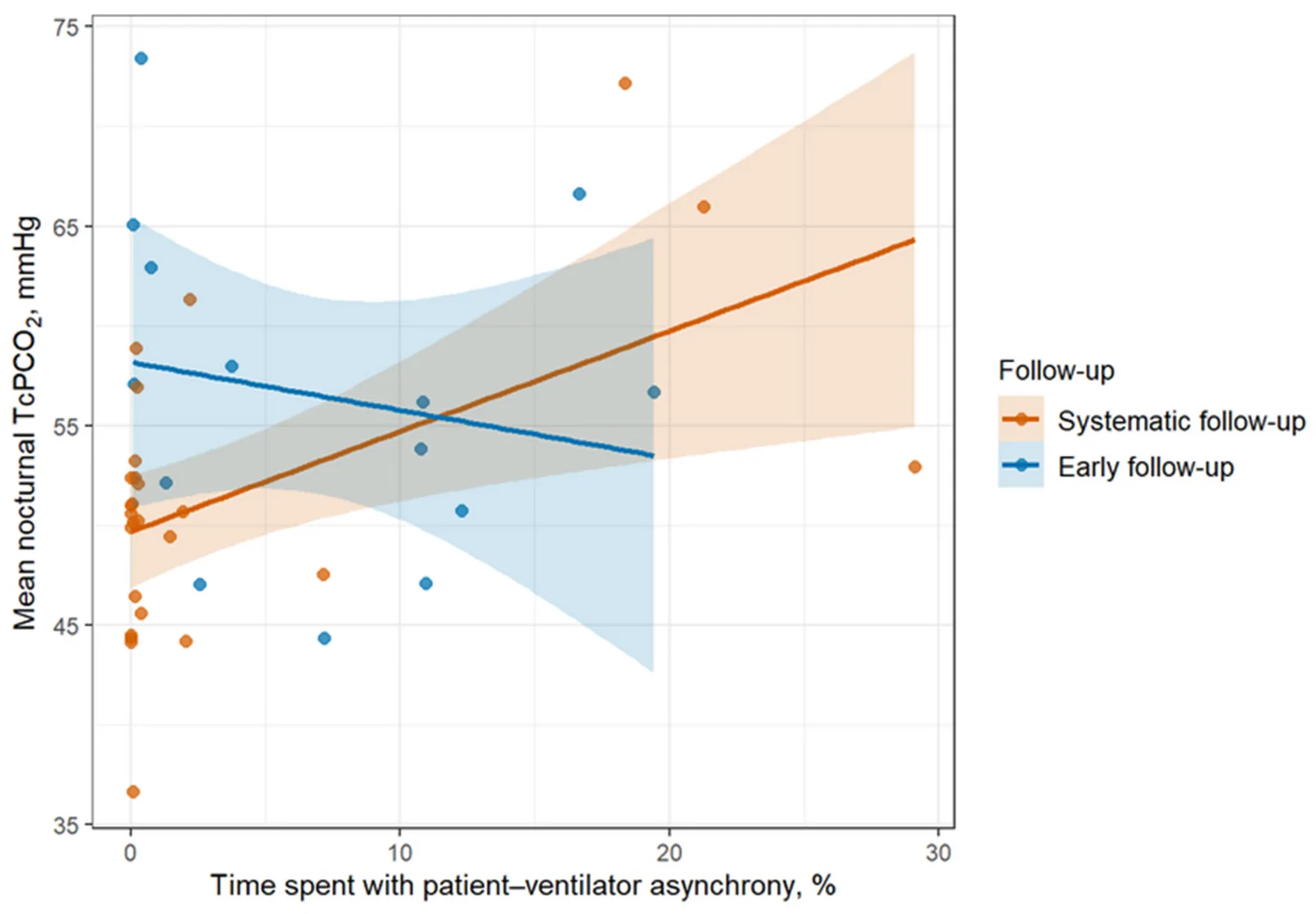
Association between patient–ventilator asynchrony (PVA) burden and nocturnal TcPCO_2_ according to follow-up timing. Lines represent linear regression fits with 95% confidence intervals. Mean nocturnal TcPCO_2_ was available in 40 patients (26 during long-term follow-up and 14 during early follow-up).

No significant interaction was observed between PVA burden and follow-up category for post-NIV PaCO_2_ or mean nocturnal SpO_2_ (Table 4). In contrast, a significant interaction was identified for mean nocturnal TcPCO_2_. Compared with long-term follow-up, the association between PVA burden and nocturnal hypercapnia was significantly attenuated during early follow-up assessments (interaction β = −1.04, 95% CI −1.68 to −0.40; *p* = 0.003) (Figure 1). This interaction was reduced after adjustment for daytime PaCO_2_ (β = −0.55, 95% CI −1.12 to 0.03; *p* = 0.076).

Stratified analyses performed in patients undergoing long-term follow-up demonstrated independent associations between PVA burden and both post-NIV PaCO_2_ (β = 0.38, 95% CI 0.04–0.72; *p* = 0.034) and mean nocturnal TcPCO_2_ (β = 0.80, 95% CI 0.51–1.08; *p* < 0.001) (Table 5). No significant association was observed for mean nocturnal SpO_2_ (β = 0.15, 95% CI −0.06 to 0.37; *p* = 0.176). Adjusted TcPCO_2_ estimates were not reported for the early acute and early planned follow-up groups because only seven patients in each group had available TcPCO_2_ data.

Taken together, these findings indicate that acute NIV initiation identifies a subgroup of patients with a greater burden of residual patient–ventilator asynchronies, particularly ineffective efforts. However, the association between PVA burden and nocturnal hypercapnia was observed primarily during long-term NIV follow-up rather than during the early post-initiation period.

## 4. Discussion

The present study provides two complementary insights into the behaviour and clinical significance of patient–ventilator asynchronies during long-term NIV. First, although no statistically significant differences in PVA, upper-airway obstruction or leak burden were detected across disease categories, patients evaluated after acute NIV initiation exhibited a substantially greater burden of residual asynchrony than those initiated electively. This difference was mainly accounted for by ineffective efforts. Second, despite this increased burden of residual asynchrony, the association between PVA and nocturnal hypercapnia was observed primarily during long-term follow-up.

The absence of significant differences in overall PVA burden across disease categories is noteworthy. Previous studies have largely focused on specific populations, including COPD, neuromuscular disorders or mixed chronic hypoventilation cohorts [6,7,8,10]. Direct comparisons of PVA burden across major etiologies of CRF are scarce. To our knowledge, no previous adult study has simultaneously compared the burden of specific PVA subtypes across the major etiologies of CRF while also considering the context of NIV initiation. Our findings suggest that residual patient–ventilator asynchronies are a common feature of long-term NIV. No statistically significant differences in residual PVA burden were detected across disease categories, although limited subgroup sizes preclude conclusions of equivalence or independence from underlying disease.

These findings extend the observations of our primary study, which identified PVA as an independent determinant of impaired gas exchange during long-term NIV. The present analysis suggests that the burden of asynchrony and its physiological consequences should be considered separately. Patients assessed shortly after NIV initiation, particularly following acute respiratory failure, may exhibit a high burden of ineffective efforts, yet these events were not associated with impaired nocturnal gas exchange. Conversely, during long-term NIV, even moderate levels of persistent asynchrony appear closely associated with residual nocturnal hypercapnia. Residual respiratory events should therefore not be interpreted in isolation. Their clinical relevance depends on the broader treatment response, including symptoms, adherence, daytime and nocturnal gas exchange, and the underlying indication for NIV.

The greater burden of residual asynchrony observed during early follow-up after acute NIV initiation is also consistent with previous studies showing that patient–ventilator asynchronies are particularly frequent during acute respiratory failure [3,4,11]. In these settings, PVAs have been largely attributed to excessive leaks, suboptimal trigger and cycling settings, and the profound respiratory instability associated with acute illness. Our findings suggest that patients initiated on NIV during acute respiratory failure remain more prone to patient–ventilator asynchronies even after clinical stabilization. However, unlike during acute respiratory failure, all recordings in the present study were obtained during scheduled follow-up in a dedicated ventilation unit, leak burden and ventilator settings were broadly similar between groups, and patients were managed according to the same local clinical practices. Therefore, the mechanisms classically proposed to explain PVA during acute NIV are unlikely to fully account for our findings. Although the underlying mechanisms remain uncertain, persistent abnormalities in respiratory drive, incomplete physiological recovery from the precipitating illness, or slower adaptation to long-term NIV may contribute to the persistence of ineffective efforts during the first months of treatment. However, the physiological consequences of PVA depend on ventilator mode, level of assistance, interface, respiratory drive, and the type of asynchrony.

Interestingly, this increased burden of asynchrony was not associated with a stronger relationship between PVA and gas exchange. Patients undergoing early follow-up remained substantially more hypercapnic than those evaluated during long-term follow-up despite broadly similar ventilator settings. This finding suggests that, shortly after NIV initiation, carbon dioxide control is influenced by multiple factors beyond patient–ventilator interaction alone, including the underlying respiratory disorder and its ongoing recovery or the ongoing adaptation to NIV [2,14]. By contrast, during established long-term NIV, when these influences have largely stabilized, persistent PVA may become a more important determinant of residual nocturnal hypercapnia. However, the interaction was attenuated after adjustment for daytime PaCO_2_, suggesting that differences in ventilatory status may partly account for the original finding. Residual confounding by age, sex, disease severity, and other unmeasured factors cannot be excluded. Moreover, the long-term group represents a selected population of patients who survived and remained on NIV for a mean of 38.2 months, rather than the longitudinal continuation of the early groups. This selection may also contribute to the observed association. More broadly, in both early and long-term NIV, gas exchange may also be influenced by ventilator settings and interface choice, which may itself depend on patient tolerance and adherence. These factors were not fully accounted for in the present analysis and therefore limit causal attribution of residual hypercapnia to PVA.

Our findings may help reconcile apparently conflicting observations from previous studies. Aarrestad et al. reported no significant association between residual respiratory events and gas exchange during long-term NIV [10], whereas Ramsay et al. demonstrated a relationship between patient–ventilator interaction and nocturnal ventilation during NIV set-up [9]. Rather than representing contradictory observations, these studies may have evaluated patients at different stages of NIV treatment. Our results suggest that the physiological consequences of residual PVA are dynamic. Similar observations have been reported in pediatric NIV, where residual respiratory events and patient–ventilator asynchronies remain frequent and may have measurable physiological consequences [11,12].

Several limitations should be acknowledged. First, this was a retrospective secondary analysis, and some subgroup analyses were limited by small sample sizes. Second, TcPCO_2_ was available in only 40 patients and was collected as part of routine clinical practice. Although measured characteristics did not differ significantly according to TcPCO_2_ availability, selection bias cannot be excluded, and estimates in the two early subgroups, each including only seven patients, should be considered exploratory. Third, the long-term follow-up group was evaluated a mean of 38 months after NIV initiation and therefore likely represents a selected population of patients who not only survived long-term but also remained on effective NIV, excluding patients in whom NIV was discontinued, switched to CPAP, or proved ineffective. Ventilator settings were individually titrated to treat chronic hypercapnic respiratory failure, rather than solely to normalize residual obstructive events, in accordance with contemporary guideline-based management [14,15,16]. Our findings cannot be extrapolated to continuous full-support NVS or very-high-pressure strategies, whose efficacy and safety were not assessed in this heterogeneous population.

Taken together with the findings of our primary study, these observations suggest that patients undergoing early follow-up after NIV initiation during acute respiratory failure exhibited a greater burden of residual PVA than those undergoing early follow-up after planned NIV initiation, predominantly in the form of ineffective efforts. However, their association with nocturnal hypercapnia was observed primarily during stable long-term NIV follow-up. Although the small early subgroups preclude excluding a similar association during early follow-up, this pattern suggests that the clinical relevance of residual patient–ventilator asynchronies may vary throughout the course of NIV treatment. These results support systematic assessment of residual respiratory events throughout NIV follow-up and provide a rationale for future interventional studies evaluating whether correction of persistent asynchronies can improve long-term ventilatory efficacy.

## Figures and Tables

**Table 1:** Baseline characteristics according to follow-up category.

Characteristic	Early Acute Follow-Up (*n* = 30)	Early Follow-Up After Planned Initiation (*n* = 19)	Long-Term Follow-Up (*n* = 74)	*p*-Value	Missing, *n* (%)
Age, years	71.1 ± 14.5	66.8 ± 12.8	63.2 ± 17.7	0.054 ^1^	0 (0.0)
Female sex, *n* (%)	22 (73.3)	10 (52.6)	34 (45.9)	0.040 ^2^	0 (0.0)
BMI, kg·m^−2^	30.3 ± 13.0	32.3 ± 8.1	31.8 ± 10.2	0.62 ^1^	0 (0.0)
Underlying disease, *n* (%)				0.64 ^3^	0 (0.0)
• COPD	11 (36.7)	6 (31.6)	28 (37.8)		
• NMD	5 (16.7)	7 (36.8)	22 (29.7)		
• OHS	10 (33.3)	5 (26.3)	15 (20.3)		
• RTD	4 (13.3)	1 (5.3)	9 (12.2)		
OSA, *n* (%)	10 (33.3)	11 (57.9)	37 (50.0)	0.23 ^2^	0 (0.0)
LTOT, *n* (%)	18 (60.0)	5 (26.3)	41 (55.4)	0.047 ^2^	0 (0.0)
FEV_1_, L	0.9 ± 0.5	1.2 ± 0.5	1.0 ± 0.5	0.22 ^1^	29 (23.6)
FVC, L	1.3 ± 0.6	1.8 ± 0.7	1.6 ± 0.7	0.22 ^1^	34 (27.6)
EPAP, cmH_2_O	6.8 ± 2.6	7.7 ± 3.3	6.6 ± 2.6	0.42 ^1^	0 (0.0)
Inspiratory pressure support, cmH_2_O	11.7 ± 2.5	9.9 ± 2.6	12.0 ± 3.4	0.037 ^1^	0 (0.0)
Backup respiratory rate, breaths·min^−1^	17.2 ± 2.4	16.9 ± 1.8	16.7 ± 2.0	0.42 ^1^	0 (0.0)
Interface (nasal/full-face), *n* (%)				0.24 ^2^	0 (0.0)
Chinstrap use, *n* (%)	1 (3.3)	2 (10.5)	9 (12.2)	0.54 ^3^	0 (0.0)
NIV treatment duration, months	<3	3	38.2 ± 52.1		0 (0.0)
Daytime PaCO_2_, mmHg	54.8 ± 8.7	52.8 ± 8.1	47.2 ± 8.6	<0.001 ^1^	5 (4.1)
Daytime PaO_2_, mmHg	75.0 ± 20.6	73.6 ± 27.6	73.9 ± 18.0	0.52 ^1^	5 (4.1)

Values are presented as mean ± standard deviation or number (%). ^1^ Kruskal–Wallis rank-sum test; ^2^ Pearson’s chi-squared test; ^3^ Fisher’s exact test. Inspiratory pressure support represents the difference between inspiratory and expiratory positive airway pressure (IPAP − EPAP). Abbreviations: BMI, body mass index; COPD, chronic obstructive pulmonary disease; EPAP, expiratory positive airway pressure; FEV_1_, forced expiratory volume in one second; FVC, forced vital capacity; LTOT, long-term oxygen therapy; NIV, non-invasive ventilation; NMD, neuromuscular disease; OHS, obesity hypoventilation syndrome; OSA, obstructive sleep apnea; PaCO_2_, arterial partial pressure of carbon dioxide; PaO_2_, arterial partial pressure of oxygen; RTD, restrictive thoracic disorder.

**Table 2:** Distribution of sleep-related respiratory events according to disease category.

Variable	COPD	NMD	OHS	RTD	Overall	*p*-Value
Leaks, % recording time	2.9 (5.5)	1.8 (4.5)	6.2 (12.8)	6.6 (14.0)	3.8 (8.9)	0.4
Leaks, events·h^−1^	3.8 (7.8)	2.1 (4.3)	6.5 (12.9)	4.8 (8.3)	4.1 (8.7)	0.5
Upper-airway obstruction, % recording time	5.3 (7.4)	10.1 (12.4)	9.7 (11.9)	6.7 (7.9)	7.9 (10.3)	0.2
Upper-airway obstruction, events·h^−1^	9.6 (13.2)	17.8 (19.5)	16.7 (18.6)	11.7 (10.9)	13.8 (16.5)	0.14
Total PVA, % recording time	3.1 (5.1)	3.3 (5.2)	5.0 (7.0)	7.5 (8.9)	4.1 (6.3)	0.14
Total PVA, events·h^−1^	3.7 (5.9)	5.0 (8.5)	6.1 (8.5)	8.6 (10.1)	5.2 (7.9)	0.2
Ineffective efforts, % recording time	2.6 (5.0)	2.2 (4.7)	3.6 (6.2)	6.6 (8.1)	3.2 (5.7)	0.23
Ineffective efforts, events·h^−1^	3.2 (5.9)	2.6 (5.1)	4.5 (7.2)	8.0 (9.6)	3.9 (6.7)	0.33
Double triggering, % recording time	0.4 (1.7)	0.3 (0.6)	1.3 (3.0)	0.2 (0.6)	0.6 (1.9)	0.33
Double triggering, events·h^−1^	0.2 (0.7)	0.7 (1.8)	1.4 (3.3)	0.2 (0.4)	0.7 (2.0)	0.23
Autotriggering, % recording time	0.1 (0.6)	0.7 (2.7)	0.1 (0.3)	0.7 (1.6)	0.4 (1.6)	0.103
Autotriggering, events·h^−1^	0.2 (0.9)	1.6 (6.4)	0.2 (0.5)	0.4 (0.6)	0.6 (3.4)	0.33

Values are mean (SD). *p*-values were obtained using Kruskal–Wallis rank-sum tests. There were no missing data for the respiratory-event variables included in this table. COPD, chronic obstructive pulmonary disease; NMD, neuromuscular disease; OHS, obesity hypoventilation syndrome; RTD, restrictive thoracic disorder.

**Table 3:** Residual sleep-related respiratory events according to follow-up context.

Variable	Long-Term Follow-Up ^1^	Early Planned Follow-Up ^1^	Early Acute Follow-Up ^1^	Overall ^1^	*p*-Value ^2^
Leaks, % recording time	3.6 (8.4)	4.7 (13.3)	3.7 (6.8)	3.8 (8.9)	0.5
Leaks, events/h	3.6 (7.4)	5.5 (13.0)	4.4 (8.8)	4.1 (8.7)	0.5
Upper-airway obstruction, % recording time	6.5 (8.9)	7.2 (9.6)	11.6 (13.0)	7.9 (10.3)	0.14
Upper-airway obstruction, events/h	11.5 (14.1)	12.7 (15.3)	20.3 (21.2)	13.8 (16.5)	0.1
Total PVA, % recording time	3.7 (6.9)	3.4 (4.2)	5.7 (5.6)	4.1 (6.3)	0.006
Total PVA, events/h	4.0 (7.5)	3.9 (4.7)	8.9 (9.4)	5.2 (7.9)	0.007
Ineffective efforts, % recording time	3.0 (6.5)	1.4 (1.8)	4.9 (5.0)	3.2 (5.7)	0.006
Ineffective efforts, events/h	3.1 (6.3)	1.8 (2.9)	7.2 (8.2)	3.9 (6.7)	0.011
Double triggering, % recording time	0.4 (1.3)	1.3 (3.1)	0.6 (2.0)	0.6 (1.9)	0.3
Double triggering, events/h	0.4 (1.2)	0.8 (1.6)	1.2 (3.2)	0.7 (2.0)	0.3
Autotriggering, % recording time	0.3 (1.6)	0.8 (2.3)	0.2 (0.7)	0.4 (1.6)	<0.001
Autotriggering, events/h	0.6 (3.9)	1.3 (3.9)	0.4 (1.1)	0.6 (3.4)	<0.001

Abbreviations: PVA, patient–ventilator asynchrony. ^1^ Values are mean (SD). ^2^ Kruskal–Wallis rank-sum test.

**Table 4:** Effect modification of the association between patient–ventilator asynchrony burden and gas exchange according to follow-up timing.

Outcome	Comparison	Interaction β (95% CI)	*p*-Value
Post-NIV PaCO_2_	Early acute vs. long-term follow-up	0.09 (−0.56 to 0.74)	0.783
Post-NIV PaCO_2_	Early planned vs. long-term follow-up	−0.39 (−1.37 to 0.58)	0.430
Mean nocturnal TcPCO_2_	All early vs. long-term follow-up	−1.04 (−1.68 to −0.40)	0.003
Mean nocturnal TcPCO_2_	All early vs. long-term follow-up, additionally adjusted for daytime PaCO_2_	−0.55 (−1.12 to 0.03)	0.076
Mean nocturnal TcPCO_2_	Early acute vs. long-term follow-up	−0.91 (−1.87 to 0.06)	0.075
Mean nocturnal TcPCO_2_	Early planned vs. long-term follow-up	−1.29 (−2.24 to −0.34)	0.012
Mean nocturnal SpO_2_	Early acute vs. long-term follow-up	−0.04 (−0.37 to 0.30)	0.837
Mean nocturnal SpO_2_	Early planned vs. long-term follow-up	0.16 (−0.21 to 0.54)	0.397

Generalized linear regression models with interaction terms. Multivariable linear regression models included PVA burden, follow-up category, their interaction, underlying disease, upper-airway obstruction, and leaks. “All early follow-up” combined the early acute and early planned groups. The daytime PaCO_2_-adjusted sensitivity analysis included 37 patients, whereas the original TcPCO_2_ interaction models included 40 patients. Analyses of mean nocturnal SpO_2_ excluded patients receiving supplemental oxygen. NIV, non-invasive ventilation; PaCO_2_, arterial carbon dioxide tension; SpO_2_, peripheral oxygen saturation; TcPCO_2_, transcutaneous carbon dioxide tension.

**Table 5:** Stratified multivariable associations between patient–ventilator asynchrony burden and gas exchange outcomes.

Outcome	Follow-Up Group	β (95% CI)	*p*
PaCO_2_	Long-term	0.38 (0.04–0.72)	0.034
PaCO_2_	Early acute	0.49 (−0.28–1.26)	0.224
PaCO_2_	Early planned CRF	0.12 (−0.92–1.16)	0.825
TcPCO_2_	Long-term	0.80 (0.51–1.08) *	<0.001
TcPCO_2_	Early acute	**	
TcPCO_2_	Early planned CRF	**	
SpO_2_	Long-term	0.15 (−0.06–0.37)	0.176
SpO_2_	Early acute	0.23 (−0.11–0.57)	0.224
SpO_2_	Early planned CRF	0.15 (−0.29–0.60)	0.522

Stratified multivariable models were performed separately within each follow-up group. β represents the change in the outcome associated with a 1% increase in recording time spent with patient–ventilator asynchrony. Models were adjusted for disease category, upper-airway obstructive events and unintentional leaks. * based on 26 patients, is reported as exploratory. ** not reported for the early acute and early planned follow-up groups because each included only seven patients, resulting in insufficient residual degrees of freedom for reliable multivariable estimation.

## Data Availability

De-identified data may be made available by the corresponding author upon reasonable request, subject to applicable ethical and data-protection requirements.

## Supplementary Materials

The following supporting information can be downloaded at: https://www.mdpi.com/article/10.3390/jcm15197695/s1, Table S1: Comparison of patients with and without available nocturnal transcutaneous carbon dioxide measurements.
