# Supplementary material for: Residual Sleep-Related Respiratory Events During Early Versus Long-Term NIV Follow-Up: Burden and Relationship with Gas Exchange
Source: J Clin Med. 2026 Oct 4;15(19):7695. doi: 10.3390/jcm15197695 (PMC13665768; doi:10.3390/jcm15197695)
Supplement: Supplementary file 1 [file jcm-15-07695-s001.zip › jcm-4523733-supplementary.pdf]

**Supplementary Table S1.** Comparison of patients with and without available nocturnal transcutaneous carbon dioxide measurements.

| **Characteristic** | **$TcPCO_2$ unavailable (n=83)** | **$TcPCO_2$ available (n=40)** | ***p*-value** |
|---|---|---|---|
| Follow-up category | | | 0.5 |
| Long-term follow-up | 48 (57.8%) | 26 (65.0%) | |
| Early acute follow-up | 23 (27.7%) | 7 (17.5%) | |
| Early planned follow-up | 12 (14.5%) | 7 (17.5%) | |
| Age, years | 66.2 ± 17.3 | 64.7 ± 15.1 | 0.3 |
| Female sex | 45 (54.2%) | 21 (52.5%) | >0.90 |
| BMI, $kg \cdot m^{-2}$ | 31.2 ± 11.4 | 32.2 ± 9.0 | 0.5 |
| Underlying disease | | | 0.3 |
| COPD | 26 (31.3%) | 19 (47.5%) | |
| NMD | 25 (30.1%) | 9 (22.5%) | |
| OHS | 23 (27.7%) | 7 (17.5%) | |
| RTD | 9 (10.8%) | 5 (12.5%) | |
| Obstructive sleep apnea | 44 (53.0%) | 14 (35.0%) | 0.083 |
| Long-term oxygen therapy | 44 (53.0%) | 20 (50.0%) | 0.8 |
| EPAP, $cmH_2O$ | 6.9 ± 2.8 | 6.6 ± 2.6 | 0.6 |
| Inspiratory pressure support, $cmH_2O$ | 11.3 ± 3.3 | 12.2 ± 2.6 | 0.14 |
| Backup respiratory rate, $breaths \cdot min^{-1}$ | 16.7 ± 2.2 | 17.3 ± 1.9 | 0.1 |
| Daytime $PaCO_2$, mmHg | 49.5 ± 9.2 | 50.8 ± 9.2 | 0.6 |
| Post-NIV $PaCO_2$, mmHg | 49.8 ± 8.7 | 50.6 ± 10.4 | >0.90 |
| PVA, % recording time | 3.9 ± 5.7 | 4.6 ± 7.4 | 0.8 |
| Upper-airway obstruction, % recording time | 8.6 ± 11.5 | 6.3 ± 7.0 | >0.90 |
| Leaks, % recording time | 3.9 ± 9.7 | 3.6 ± 7.2 | 0.3 |

Data are presented as mean ± standard deviation or n (%). Continuous variables were compared using the Wilcoxon rank-sum test and categorical variables using Fisher's exact test. Daytime $PaCO_2$ was missing in five patients and post-NIV $PaCO_2$ in one patient.

BMI, body mass index; COPD, chronic obstructive pulmonary disease; EPAP, expiratory positive airway pressure; LTOT, long-term oxygen therapy; NIV, non-invasive ventilation; NMD, neuromuscular disease; OHS, obesity hypoventilation syndrome; PVA, patient–ventilator asynchrony; RTD, restrictive thoracic disorder; $SpO_2$, peripheral oxygen saturation; $TcPCO_2$, transcutaneous carbon dioxide tension.
